# A systematic review of the role of videolaryngoscopy in successful orotracheal intubation

**DOI:** 10.1186/1471-2253-12-32

**Published:** 2012-12-14

**Authors:** David W Healy, Oana Maties, David Hovord, Sachin Kheterpal

**Affiliations:** 1 Department of Anesthesiology, University of Michigan Hospital, 1500 East Medical Center Drive 1H247, Box 0048, Ann Arbor, Michigan 48109, USA

**Keywords:** Laryngoscopy, Airway management, Intubation, Technology

## Abstract

**Background:**

The purpose of our study was to organize the literature regarding the efficacy of modern videolaryngoscopes in oral endotracheal intubation, then perform a quality assessment according to recommended external criteria and make recommendations for use.

**Methods:**

Inclusion criteria included devices with recent studies of human subjects. A total of 980 articles were returned in the initial search and 65 additional items were identified using cited references. After exclusion of articles failing to meet study criteria, 77 articles remained. Data were extracted according to the rate of successful intubation and improvement of glottic view compared with direct laryngoscopy. Studies were classified according to whether they primarily examined subjects with normal airways, possessing risk factors for difficult direct laryngoscopy, or following difficult or failed direct laryngoscopy.

**Results:**

The evidence of efficacy for videolaryngoscopy in the difficult airway is limited. What evidence exists is both randomized prospective and observational in nature, requiring a scheme that evaluates both forms and allows recommendations to be made.

**Conclusions:**

In patients at higher risk of difficult laryngoscopy we recommend the use of the Airtraq, CTrach, GlideScope, Pentax AWS and V-MAC to achieve successful intubation. In difficult direct laryngoscopy (C&L >/= 3) we cautiously recommend the use of the Airtraq, Bonfils, Bullard, CTrach, GlideScope, and Pentax AWS, by an operator with reasonable prior experience, to achieve successful intubation when used in accordance with the ASA practice guidelines for management of the difficult airway. There is additional evidence to support the use of the Airtraq, Bonfils, CTrach, GlideScope, McGrath, and Pentax AWS following failed intubation via direct laryngoscopy to achieve successful intubation. Future investigation would benefit from precise qualification of the subjects under study, and an improvement in overall methodology to include randomization and blinding.

## Background

Since Macintosh [1] (1943) and Miller [2] (1941) envisioned and developed their direct laryngoscopes attempts have been made to improve on these techniques and equipment using technological advances. Nevertheless, these original techniques have withstood the test of time and remain the mainstay of intubation globally. Direct laryngoscopy (DL) relies on the formation of a “line-of-sight” between the operator and the laryngeal inlet, success reliant on careful head positioning and consistent anatomy. When these conditions are not met, for example in poor tissue mobility, limited mouth opening, or enlarged tongue, the failure rate of intubation with conventional direct laryngoscopy increases [3-5].

Videolaryngoscopy (VL) is a relatively recent development that attempts to improve the success of tracheal intubation. High-resolution micro cameras and small portable flat-screen monitors are used in an attempt to improve upon the view and success rate of direct laryngoscopy. Similar technologies have been successfully applied to other fields of medicine such as laparoscopic and robotic surgery, making new techniques and procedures possible [6]. The use of Videolaryngoscopy produces a view of the laryngeal inlet independent of the line of sight, particularly when an angulated device is used. This may free them from some of the conditions essential to the success of direct laryngoscopy. There is an assumption that improved lighting and a better view can improve the success of laryngoscopy. This may be incorrect as an improvement in success may be limited by both use of unfamiliar equipment and difficulty placing an endotracheal tube out of the line of sight. Some previous reviews have indicated an advantage when using videolaryngoscopy [7-9] but a need remains for an systematic evidence based review of the efficacy of videolaryngoscopy above that of direct laryngoscopy.

To appreciate any benefit from the use of videolaryngoscopy we need to appreciate the mechanism and incidence of failure of direct laryngoscopy. The incidence of difficulty encountered during direct laryngoscopy is difficult to ascertain as it depends upon both definition and patient selection. The best evidence available is from a meta-analysis of 50,760 patients in which difficulty at laryngoscopy occurred in 5.8% (95% CI 4.5 – 7.5) of subjects [10]. Of note, the metanalysis did exclude all patients whose airways were “anatomically abnormal” or in whom DL was thought inappropriate. The definition of difficult laryngoscopy was broad and included all subjects with Cormack and Lehane views 3 or greater. The actual incidence of difficult intubation in this difficult laryngoscopy group is presumably less than 5.8% as many patients with a Cormack and Lehane view 3 can successfully be intubated with direct laryngoscopy and the use of a gum elastic bougie by a reasonably experience practitioner. However, even given this broad definition of difficult laryngoscopy, this still suggests an impressive overall intubation success rate of >95% [11] for direct laryngoscopy among patients thought to be suitable for this technique. It is this high standard against which the new methods of videolaryngoscopy must be assessed.

## Methods

Pubmed and Cochrane review searches were made of all published articles regarding Video Laryngoscopy (VL) from 1999 to April 2011. The following search terms were used: Airtraq, Berci DCI, Bonfils Fibre(er) scope, Bonfils Intubation, Bullard laryngoscope, C-MAC, C-MAC D-blade, CTrach, video laryngoscopy, EVO videolaryngoscope, GlideScope, Glidescope Direct, LMA CTrach, McGrath laryngoscope, McGrath MAC, McGrath series 5, Pentax Airway Scope, Pentax AWS, Rusch, Shikani, Storz Berci, Storz CMAC, Styletscope, V-MAC, Upsherscope, WuScope. X-Lite. Additional search items, for classification purposes, were cervical, cervical limitation, cervical stabilization, obesity, difficult intubation, failed intubation, failed ventilation and education. From this selection all articles were reviewed including randomized controlled trials, observational studies, review articles, meta-analyses and editorials. Bibliographies were checked manually for any relevant articles. Articles published in the ASA meeting proceedings were included and a search made of all ongoing clinical trials in Clinicaltrials.gov.

### Inclusion criteria

Orotracheal intubation.

Procedure performed by trained operators.

English language or accessible translation of key outcomes and methodology.

Device had at least 10 articles published on its use during the previous 5 years (until April 2011).

### Exclusion criteria

Studies of patients aged less than 18 years.

Duplicates, unrelated studies, abstracts, single case reports and small studies (less than 5 subjects).

Manikin studies.

### Data extraction

To summarize the data available from the multiple studies the following measures were extracted from each article:

An overall measure of study quality (based on SIGN recommendations [12]).

Study device.

Summary of method.

Number of subjects in study group.

Number of predicted normal airway (eg MP 1/2).

Number of predicted difficult (eg MP 3/4).

Number of difficult direct laryngoscopies (known or C&L >3 on Mac DL).

Improvement in laryngeal view compared with direct laryngoscopy.

Average time to intubation. Expressed as central tendency (Mean or median) and variability (95% confidence interval or Inter Quartile Range).

Success rate (percentage) on 1st attempt and overall success (OA).

## Results

**Figure 1:**
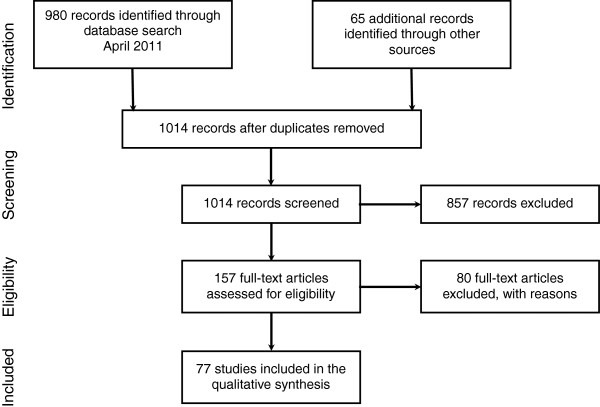
Information flow through the systematic review.

A total of 980 articles were returned in the initial Pub Med search and 65 additional items were identified using references cited in these articles. After exclusion of articles failing to meet study criteria, 77 articles remained (Figure 1, Tables 1, 2 and 3).

**Table 1:** Data extraction

**1st Author**	**Quality**	**Device**	**Method**	**N device**	**Predicted easy (MP 1-2)**	**Predicted difficult (MP 3-4)**	**Difficult laryngoscopy (C&L 3-4 on DL)**	**Achievement of C&L I view**	**Time to intubation 95% CI or IQR**	**Success% 1st attempt Overall (OA)**
Maharaj (2006) [13]	(+)	Airtraq	Randomized, 60 subjects, Airtraq v Mac DL	30	30	0	No data	95%	Mean 12.2	100% 1st
								Mac DL 70%	(95% CI 9.1 to 15.3)	100% OA
Maharaj (2007) [14]	(+)	Airtraq	Randomized, 40 subjects, Airtraq v Mac DL, Cervical spine limitation (MILS)	20	20	0	No data	95%	Mean 13.2	No data
								Mac DL 30%	(95% CI 10.6 to 15.7)	
Maharaj (2007) [15]	na	Airtraq	Observational, Case series, 7 subjects, failed Mac DL	7	0	7	4 C&L 4	100%	Mean 14	No data
								Mac DL 0%	(95% CI 8.5 to 18.9)	
Ndoko (2007) [16]	(-)	Airtraq	Randomized, 70 subjects, Mac DL v Airtraq, risk of difficulty	35	0	35	No data	No data	Mean 30	100% OA
									(95% CI 21.4 to 35.8)	
Arslan (2009) [17]	(+)	Airtraq	Randomized, 86 subjects, Airtraq v CTrach, Cervical spine limitation (collar)	43	42	1	No data	No data	Mean 25.6	No data
									(95% CI 21.4 to 29.8)	
Dhonneur (2009) [18]	(+)	Airtraq	Randomized, 318 subjects, Airtraq v Mac DL v CTrach, obese	106	82	24	No data	94%	Mean 29	No data
								Mac DL 51%	(95% CI 26.7 to 31.3)	
Lange (2009) [19]	(+)	Airtraq	Randomized, 60 subjects, Mac DL then Airtraq v GlideScope	30	26	4	4 C&L 3-4	90%	Mean 19.7	No data
								Mac DL 57%	(95% CI to 15.7 to 23.8)	
Malin (2009) [20]	na	Airtraq	Observational, Case series, 47 subjects, failed Mac DL	47	0	47	47 C&L 2b-4	85%	No data	95% 1st
								Mac DL 0%		100%OA
Turkstra (2009) [21]	(+)	Airtraq	Randomized, cross-over,24 subjects, Airtraq v Mac, cervical spine limitation (MILS)	24	24	0	2	90%	Median 8.8	100% 1st
								Mac DL 20%	(IQR 6.7 to 10.6)	100% OA
Chalkeidis (2010) [22]	(+)	Airtraq	Randomized, 63 subjects, Airtraq v Mac DL	35	25	10	No data	No data	Mean 30	80% OA
									(95% CI 27.1 to 32.9)	
Koh (2010) [23]	(+)	Airtraq	Randomized, 50 subjects, Airtraq v Mac DL, Cervical spine limitation (collar)	25	20	5	No data	No data	Mean 50	100% OA
									(95% CI to 36.2 to 63.8)	
Halligan (2003) [24]	na	Bonfils	Observational, Case series, 60 subjects	60	58	2	No data	No data	Median 33	98% OA
									(IQR 24 to 50)	
Wong (2003) [25]	na	Bonfils	Observational, Case series, 36 subjects	36	No data	No data	No data	No data	Median 80	86% OA
									(No IQR report)	
Bein (2004) [26]	(-)	Bonfils	Randomized, 80 subjects, Bonfils v ILMA, Risk of difficulty	40	12	28	No data	No data	Median 40	98% 1st
									(IQR 23 to 77)	100% OA
Bein (2004) [27]	na	Bonfils	Observational, Case series, 25 subjects, failed Mac DL	25	0	No data	25	No data	Median 48	No data
									(IQR 30 to 80)	
Wahlen (2004) [28]	(-)	Bonfils	Randomized, 48 subjects, Bonfils v Mac DL v Bullard v ILMA	12	12	0	No data	No data	Mean 52	92% OA
									(95% CI 38.1 to 66.1)	
Byhahn (2008) [29]	(+)	Bonfils	Randomized, 76 subjects, Bonfils v Mac DL, Cervical spine limitation (collar)	38	38	0	Mac group 17	82%	Mean 64	71% 1st
								Mac DL 5%	(95% CI 56.1 to 71.9)	82% OA
Corbanese (2009) [30]	na	Bonfils	Observational, Case series, 100 subjects	100	100	0	No data	No data	Median 30	89% 1st
									(IQR 25 to 40)	98% OA
Corso (2010) [31]	na	Bonfils	Observational, Case series, 10 subjects	10	No data	No data	No data	No data	No data	No data
MacQuarrie (1999)[32]	na	Bullard	Observational, Case series, 80 subjects, Cervical spine limitation (collar)	40 x 2 grps	28	No data	52	No data	MFIS group	89% 1st
									Mean 41	98% OA
									(95% CI 35.3 to 46.7)	
									ISETT group	
									Mean 45.4 (95% CI 39.4 to 51.4)	
Shulman (2001) [33]	(-)	Bullard	Randomized, cross-over, 50 subjects, Bullard v FOI, Cervical spine limitation (MILS)	25 x 2 grps	No data	No data	No data	No data	Standard Group: Mean 37	85% OA
									(95% CI 26.2 to 47.8)	
									Cricoid Group	
									Mean 38 (95% CI 26.9 to 49.1)	
Wahlen (2004) [28]	(+)	Bullard	Randomized, 48 subjects, Bullard v Mac v Bonfils v ILMA	12	12	0	No data	92%	Mean 16.1	92% 1st
								Mac DL 33%	(95% CI 12.1 to 20)	92%OA
Nileshwar (2007) [34]	(+)	Bullard	Randomized, 62 subjects, Mac DL then Bullard v ILMA, cervical spine limitation (MILS)	31	19	No data	12	No data	Mean 84	86% 1st
									(95% CI 66.4 to 101.6)	90% OA
Teoh (2010) [35]	(+)	C-MAC	Randomized, 400 subjects GlideScope v Pentax AWS v C-MAC v MacDL	100	85	15	No data	87%	Mean 31.9	93% 1st
									(95% CI 28.4 to 35.4)	100% OA
Dhonneur (2006) [36]	(+)	CTrach	Randomized, 104 subjects, Mac DL v CTrach, obese	52	43	9	No data	75%	Mean 176	No data
									(95% CI 166 to 186)	
Goldman (2006) [37]	na	CTrach	Observational, Case series, 328 subjects	328	No data	No data	No data	91%	No data	No data
Goldman (2006) [38]	na	CTrach	Observational, Case series, 6 subjects	6	3	3	6	100%	No data	No data
								Mac DL 0%		
Liu (2006) [39]	na	CTrach	Observational, Case series, 100 subjects	100	84	26	9	28%	Median 166	No data
								Mac DL 59%	(IQR 114 to 233)	
Timmerman (2006) [40]	na	CTrach	Observational, Case series, 10 subjects	10	No data	No data	No data	30%	No data	No data
Timmerman (2006) [41]	na	CTrach	Observational, Case series, 60 subjects	60	No data	No data	3	55%	No data	No data
Cattano (2007) [42]	na	CTrach	Observational, Case series, 15 subjects, obese	15	No data	No data	No data	60%	No data	No data
Dhonneur (2007) [43]	(+)	CTrach	Randomized, 120 subjects, CTrach v MacDL	60	No data	No data	No data	93%	Mean 119	No data
									(95% CI 107.6 to 130.4)	
Ng (2007) [44]	(-)	CTrach	Randomized trial, 106 subjects, CTrach v GlideScope	54	54	0	No data	85%	Mean 73	No data
									(95% CI 63.2 to 82.8)	
Liu (2008) [45]	(+)	CTrach	Randomized, 271 subjects, CTrach v ILMA (Fastrach)	134	118	16	13	93%	Median 116	93% 1st
								Mac DL 59%	(IQR 82 to 156)	100% OA
Nickel (2008) [46]	na	CTrach	Observational, Case series, 16 subjects	16	No data	No data	No data	44%	No data	No data
Arslan (2009) [17]	(+)	CTrach	Randomized, 86 subjects, Airtraq v CTrach, Cervical spine limitation (collar)	43	42	1	No data	No data	Mean 66.3	93% 1st
									(95% CI 57.3 to 75.3)	100% OA
Dhonneur (2009) [18]	(+)	CTrach	Randomized, 318 subjects, Airtraq v Mac DL v CTrach, obese	106	78	28	No data	97%	Mean 109	100% OA
								Mac DL 51%,	(95% CI 103.9 to 114.1)	
Liu (2009) [47]	na	CTrach	Observational, Case series, 48 subjects	48	18	30	26	in 96%	No data	No data
								Mac DL 0%		
Malik (2009) [48]	(+)	CTrach	Randomized, 90 subjects, Pentax AWS v Mac DL v CTrach, cervical spine limitation (MILS)	30	30	0	No data	67%	Median 46	84% 1st
								Mac DL 20%	(IQR 38 to 107)	90% OA
Ng (2009) [49]	na	CTrach	Observational, Case series, 50 subjects, cervical spine limitation (MILS)	50	45	5	11	98%	No data	No data
								Mac DL 44%		
Swadia (2009) [50]	na	CTrach	Observational, Case series, 20 subjects	20	20	0	No data	60%	Mean 347.8	No data
									(95% CI 342.8 to 352.8)	
Agro (2003) [4]	na	GlideScope	Observational, Case series, 15 subjects, C spine limitation (collar)	15	No data	No data	10	33%	Mean 38	No data
								Mac DL 0%	(no SD report)	
Cooper (2005) [51]	na	GlideScope	Observational, Case series, 728 subjects	728	579	148	34/133	86%	No data	96% OA
								Mac DL 49%		
Doyle (2005) [52]	na	GlideScope	Observational, Case series, 747 subjects	747	No data	No data	No data	No data	No data	100 % OA
Hsiao (2005) [53]	na	GlideScope	Observational, Case series, 103 subjects, Mac DL then GlideScope	103	No data	No data	22	80%	No data	No data
								Mac DL 52%		
Lim (2005) [54]	(+)	GlideScope	Randomized, 60 subjects, GlideScope v Mac DL, Cervical spine limitation (MILS)	30	30	0	8 in Mac DL group	67%	Mean 41.8	86% 1st
								Mac DL 13%	(95% CI 34.2 to 49.4)	94% OA
Rai (2005) [55]	na	GlideScope	Observational, Case series, 50 subjects	50	No data	No data	1	88%	Median 40	No data
								Mac DL 44%	(IQR 30 to 55)	
Sun (2005) [56]	(+)	GlideScope	Randomized, 200 subjects, Mac DL then Mac v GlideScope	100	88	12	15	75%	Mean 46	94% 1st
								Mac DL 59%	(95% CI 42 to 49)	99% OA
Turkstra (2005) [57]	(+)	GlideScope	Randomized, cross-over, 36 subjects, Mac DL and GlideScope, cervical spine limitation (MILS)	18	16	2	No data	No data	Mean 27	No data
									(95% CI 21.0 to 33.0)	
Ng (2007) [44]	(-)	GlideScope	Randomized, 106 subjects, CTrach v GlideScope	52	52	0	No data	100 %	Mean 43	No data
									(95% CI 36.9 to 49.1)	
Xue (2007) [58]	na	GlideScope	Observational, Case series, 91 subjects	91	79	12	19/27	74%	Mean 38	97% 1st
								Mac DL 11%	(95% CI 35.7 to 40.3)	100% OA
Malik (2008) [59]	(+)	GlideScope	Randomized, 120 subjects, GlideScope v Pentax AWS v Mac DL v Truview, Cervical spine limitation (MILS)	30	30	0	No data	70%	Mean 18.9	No data
								Mac DL 20%	(95% CI 16.7 to 21.9)	
Tremblay (2008) [60]	na	GlideScope	Observational, Case series, 400 subjects, Mac DL then GlideScope	400	347	53	26	90%	Mean 21	84% 1st
								Mac DL 67%	(95% CI 19.6 to 22.4)	99% OA
Robitaille (2008) [61]	(+)	GlideScope	Randomized, cross over, 20 subjects, cervical spine limitation (MILS)	20	No data	No data	1	50%	No data	No data
								Mac DL 0%		
Bathory (2009) [62]	na	GlideScope	Observational, Case series, 50 subjects, Mac DL then GlideScope, Cervical spine limitation (MILS)	50	48	2	50	8%	Median 50	No data
								Mac DL 0%	(IQR 41-63 s)	
Stroumpoulis [63] (2009)	na	GlideScope	Observational, Case series, 112 subjects, Mac DL then GlideScope,	112	70	42	41	61%	No data	90% 1st
								Mac DL 38%		98% OA
Lange (2009) [19]	(+)	GlideScope	Randomized, 60 subjects, Mac DL then Airtraq v GlideScope	30	27	3	5	90%	Mean 17.3	97% 1st
								Mac DL 63%	(95% CI 14.8 to 19.8)	100% OA
Liu (2009) [64]	(+)	GlideScope	Randomized, 70 subjects, GlideScope v Pentax AWS), cervical spine limitation (MILS)	35	23	12	20	40%	Mean 71.9	No data
								Mac DL 20%	(95% CI 55.5 to 88.3)	
Maassen (2009) [65]	(+)	GlideScope	Randomized, 150 subjects, Mac DL then GlideScope v V-MAC v McGrath, Obese	50	37	13	17	No data	Mean 33	No data
									(95% CI 27.9 to 38.1)	
Malik (2009) [66]	(+)	GlideScope	Randomized, 75 subjects,Pentax AWS v GlideScope v Mac DL, Risk of difficulty	25	0	25	No data	88%	Median 17	88 % 1st
									(IQR 12 to 21)	96% OA
Nouruzi-Sedeh (2009) [67]	(-)	GlideScope	Randomized, 200 subjects, Mac DL v GlideScope, untrained operators	100	No data	No data	No data	66%	Mean 63	93% 1st
								Mac DL 32%	(95% CI 57.0 to 68.9)	100% OA
Teoh (2009) [68]	(-)	GlideScope	Randomized, 140 subjects, GlideScope v Pentax AWS	70	62	8	No data	81%	Median 27.8	No data
									(IQR 22 to 36)	
Turkstra (2009) [69]	(+)	GlideScope	Randomized, 80 subjects, GlideScope alone (comparing stylets)	79	67	12	No data	73%	Median 37	92% 1st
										96% OA
Van Zundert (2009)[70]	(+)	GlideScope	Randomized, 450 subjects, Mac DL then GlideScope v V-MAC v McGrath	150	134	16	No data	No data	Mean 34	No data
									(95% CI 30.8 to 37.2)	
Hirabayashi (2010) [71]	(-)	GlideScope	Randomized, 200 subjects, GlideScope v Mac DL	100	No data	No data	No data	No data	Mean 64	94% 1st
									(95% CI 57.5 to 70.5)	100% OA
Serocki (2010) [72]	(+)	GlideScope	Randomized, cross-over, 120 subjects GlideScope v V-MAC v Mac DL, Risk of difficulty	120	68	52	36	36%	Median 33	91% 1st
								Mac DL 0%	(IQR 18 to 38)	100% OA
Siu (2010) [73]	na	GlideScope	Observational, Case series, 742 subjects	742	408	256	78	62%	No data	No data
Teoh (2010) [35]	(+)	GlideScope	Randomized, 400 subjects, GlideScope v Pentax AWS v CMAC v Mac DL	100	71	29	No data	78%	Mean 31	91% 1st
									(95% CI 28.0 to 34.0)	100% OA
Aziz (2011) [74]	na	GlideScope	Observational, Case series, 2004 subjects	2004	1329	675	239 failed DL	No data	No data	No data
Shippey (2007) [75]	na	McGrath	Observational, Case series, 75 subjects	75	63	11	1	No data	Median 25	93% 1st
									(IQR 18.5 to 34.4)	98% OA
O’Leary (2008) [76]	na	McGrath	Observational, Case series, 30 subjects, failed DL	30	No data	No data	12	77%	No data	No data
								Mac DL 3%		
Maassen (2009) [65]	(+)	McGrath	Randomized, 150 subjects, Mac DL then GlideScope v V-MAC v McGrath, Obese	50	38	12	14	No data	Mean 41	8% 1st
									(95% CI 33.9 to 48.1)	100% OA
Van Zundert (2009)[70]	(+)	McGrath	Randomized, 450 subjects, Mac DL then GlideScope v V-MAC v McGrath	150	133	17	No data	No data	Mean 38	83% OA
									(95% CI 34.3 to 41.7)	
Walker (2009) [77]	(+)	McGrath	Randomized, 120 subjects, McGrath v Mac DL	60	58	2	No data	No data	Median 47	95% 1st
									(IQR 39 to 60)	100% OA
Hughes (2010) [78]	na	McGrath	Observational, Case series, 6 subjects	6	No data	No data	No data	No data	No data	No data
Noppens (2010) [79]	na	McGrath	Observational, Case series, 61 subjects, C&L 3-4 failed Mac DL	61	No data	No data	61 C&L 3-4	87%	No data	95% OA
								Mac DL 0%		
Asai (2008) [80]	na	Pentax AWS	Observational, Case series, 100 subjects	100	100	0	No data	No data	Median 35	96% 1st, 99%OA
									(No IQR report)	
Enomoto (2008) [81]	(+)	Pentax AWS	Randomized, cross-over, 203 subjects, Mac DL v Pentax AWS, cervical spine limitation (MILS)	203	194	9	22	Mac DL 61%	Mean 54	
									(95% CI 52.1 to 55.9)	
Hirabayashi (2008) [82]	na	Pentax AWS	Observational, Case series, 405 subjects	405	No data	No data	16	No data	Mean 42	95% 1st
									(95% CI 3.8 to 81)	100%OA
Malik (2008) [59]	(+)	Pentax AWS	Randomized, 120 subjects, Pentax AWS v Mac v GS v Truview, cervical spine limitation (MILS)	30	30	0	No data	97%	Mean 16.7	No data
								Mac DL 20%	(95% CI 14 to 19.4)	
Suzuki (2008) [83]	na	Pentax AWS	Observational, Case series, 320 subjects	320	265	55	46	99%	Mean 20.1	96% 1st
								Mac DL 55%	(95% CI 19 to 21.2)	100% OA
Asai (2009) [84]	na	Pentax AWS	Observational, Case series, 270 subjects, difficult Mac DLs	270	179	91	256	96%	No data	No data
								Mac DL 0%		
Hirabayashi (2009) [85]	(+)	Pentax AWS	Randomized, 520 subjects, Mac DL v Pentax AWS	264	No data	No data	No data	No data	Mean 44	96% 1st
									(95% CI 41.7 to 46.2)	100% OA
Liu (2009) [64]	(+)	Pentax AWS	Randomized, 70 subjects, Pentax AWS v GlideScope, Cervical spine limitation (MILS)	35	25	10	19	97%	Mean 34.2	100% OA
								Mac DL 19%	(95% CI 25.6 to 42.8)	
Malik (2009) [48]	(+)	Pentax AWS	Randomized, 90 subjects, Pentax AWS v Mac v CTrach, cervical spine limitation (MILS)	30	30	0	No data	100%	Median 10	93% 1st
								Mac DL 20%	(IQR 8 to 15)	100%OA
Malik (2009) [66]	(+)	Pentax AWS	Randomized, 75 subjects, Pentax AWS v GlideScope v Mac, Risk of difficulty	25	1	24	No data	100%	Median 15	72 % 1st
									(IQR 8 to 31)	100% OA
Teoh (2009) [68]	(+)	Pentax AWS	Randomized, 140 subjects, Pentax AWS v GlideScope	70	60	10	No data	98%	Median 19	87% 1st
									(IQR 14 to 4.5)	100 OA
Teoh (2010) [35]	(+)	Pentax AWS	Randomized, 400 subjects, GlideScope v Pentax AWS v C-MAC v Mac DL	100	83	17	No data	97%	Mean 20.6	95% 1st
									(95% CI 18.3 to 22.9)	100% OA
Kaplan (2006) [86]	na	V-MAC	Observational, Case series, 865 subjects, Mac DL then V-MAC	865	No data	No data	123	56%	No data	No data
								Mac DL 36%		
Cavus (2009) [87]	na	C-MAC	Observational, Case series, 60 subjects	60	42	18	No data	No data	Median 16	87% 1st
									(IQR 6 to 58)	100% OA
Jungbauer (2009) [88]	(+)	V-MAC	Randomized, 200 subjects, Mac DL v V-MAC, at risk of difficulty	100	1	99	36	45%	Mean 40	No data
								Mac DL 23%	(95% CI 33.9 to 46.1)	
Maassen (2009) [65]	(+)	V-MAC	Randomized, 150 subjects, Mac DL then GlideScope v V-MAC v McGrath, Obese	50	37	13	14	No data	Mean 17	No data
									(95% CI 15 to 19)	
Van Zundert (2009)[70]	(+)	V-MAC	Randomized, 450 subjects, Mac DL then GlideScope v V-MAC v McGrath	150	132	18	No data	No data	Mean 18	No data
									(95% CI 16.1 to 19.9)	
Meininger (2010) [89]	na	C-MAC	Observational, Case series, 94 subjects Mac DL then C-MAC	94	No data	No data	18	43%	No data	No data
								Mac DL 35%		
Serocki (2010) [72]	(++)	V-MAC	Randomized, cross-over, 120 subjects GlideScope v V-MAC v Mac DL, Risk of difficulty	120	68	52	36	31%	Median 27	No data
								Mac DL 0%	(IQR 17 to 94)	

(Refer to Table 4 for guide to quality assessment grading).

**Table 2:** Level of evidence summary

**Device**	**Outcome**	**Failed DL**	**Difficult DL (C&L >/= 3)**	**At Higher Risk of Difficult DL**	**Unselected**
**Airtraq**	**Success 1**st **attempt**	No data	No data	1+, 96-100% [23],[17],[14]	1+, 93-100% [19],[13]
	**Success Overall**	3, 80-100% [20], [15]	3, 80-100% [20], [15]	1+, 96-100% [23],[17],[18],[14],[21],[16]	1+, 89-100% [22],[19],[90],[13]
	**% C&L 1 of glottis**	3, 85- 100% [20], [15]	3, 89-100% [20], [15]	1+, Improvement, 90-95% [21],[18],[14]	1+, Improvement, 90-95% [19],[13]
	**Time to success**	No data	No data	1+, No [16],[18],[23],[21]	1+, No [13],[22]
**Bonfils**	**Success 1**st **attempt**	3, 88% [27]	3, 88% [27]	1-, 88% [29]	3, 89% [30]
				3, 88% [27]	
	**Success Overall**	3, 96% [27]	3, 96% [27]	1-, 82% [29]	1-, 86-98% [30],[25],[28],[24]
				3, 96% [27]	
	**% C&L 1 of glottis**	No data	No data	1-, Improvement, 82% [29])	No data
	**Time to success**	No data	No data	1-, No [29]	1-, No [28]
**Bullard**	**Success 1**st **attempt**	No data	3, 89% [32]	1-, 86% [34]	1-, 92% [28]
				3, 89% [32]	
	**Success Overall**	No data	3, 98% [32]	1-, 85-100% OA [34],[32],[33]	1-, 92% OA [28]
				3, 98% [32]	
	**% C&L 1 of glottis**	No data	No data	No data	1-, Improvement, 92% [28]
	**Time to success**	No data	No data	No data	1-, Yes [28]
**CTrach**	**Success 1**st **attempt**	No data	No data	1-, 84-93% [48],[49],[36],[40],[17]	1-, 67-100% [44],[91],[39],[37],[45],[43],[46],[41]
	**Success Overall**	3, 100% [38]	3, 95.8% [47]	1-, 90-100% [48],[18],[49],[42],[36],[40],[17]	1-, 93-100% [44],[91],[39],[37],[45],[43],[46],[41],[50]
	**% C&L 1 of glottis**	3, 100% [38]	3, 95.8% [47]	1-, Improvement, 30-98% [41],[48],[42],[36],[49]	1-, Improvement, 28-93% [39],[46],[41],[50],[44],[91],[45],[37],[43]
	**Time to success**	No data	No data	1-, No [36],[18],[48]	1-, No [43]
**GlideScope**	**Success 1**st **attempt**	No data	3, 90% [63]	1+ 16-93% [65],[64],[66],[54],[74],[59]	1-, 78-98% [73],[55],[56],[53],[58],[60],[44],[68],[35],[19], [71]
	**Success Overall**	3, 94% [74]	3, 98-100% [62,63]	1+, 89-100% [65],[64],[66],[54],[74],[72],[59],[61],[62]	1-, 71-100% [52],[55],[56],[53],[58],[60],[92],[67],[51],[44], [68],[35],[19],[71],[93]
	**% C&L 1 of glottis**	No data	3, Improvement, 8% [62]	1+, Improvement, 33-88% [4],[72],[64],[61],[54],[59],[66]	1-, Improvement, 62-100% ([73],[67],[58],[56],[35],[53],[68],[94],[55],[60], [19],[44]
	**Time to success**	No data	3, No [62]	1+, No [59],[72]	1-,No [71]
**McGrath**	**Success 1**st **attempt**	No data	No data	No data	1-, 93-95% [75],[77]
	**Success Overall**	3, Improvement, 83-95% [76],[79]	No data	No data	1-, 98-100% [75],[77]
	**% C&L 1 of glottis**	3, Improvement, 77--87% [76],[79]	No data	No data	No data
	**Time to success**	No data	No data	No data	1-, No [77]
**Pentax AWS**	**Success 1**st **attempt**	3, 94% [84])	3, 94% [84]	1+, 72-97% [59],[48],[95]^,^[64]	1+, 87-96% [80],[83]^,^[96],[85],[68],[35]
	**Success Overall**	3, 99% [84]	3, 99% [84]	1+, 97-100% [59],[48],[95],[64],[81]	1+, 99-100% [80],[83],[96],[85],[68],[35]
	**% C&L 1 of glottis**	3, Improvement, 96% [84]	3, Improvement, 96% [84]	1+, Improvement, 97-100% [59],[64],[48],[81],[66]	1+, Improvement, 97-99% [68],[35],[83]
	**Time to success**	No data	No data	1+, No [81],[59],[95]	1+, Yes [85],[35]
**V-MAC**	**Success 1**st **attempt**	No data	No data	1+, 64% [65]	1-, 87-93% [87],[68]
	**Success Overall**	No data	No data	1+, 98-100% [87],[65],[72],[88]	1-, 99-100% [87],[68],[89]
	**% C&L 1 of glottis**	No data	No data	1+, Improvement, 31-45% [72],[88]	1-, Improvement, 43-100% [89],[86],[35],[87]
	**Time to success**	No data	No data	1+, No [72]	1-, No [35]

**Table 3:** Level of evidence for overall success for devices under study

	**Good evidence**	**Weak evidence**	**No evidence**
	**(Level 1+)**	**(Level 3)**	
**Subjects at higher risk of difficulty during DL**	Airtraq	Bonfils	McGrath
	CTrach	Bullard	
	GlideScope		
	Pentax AWS		
	V-MAC		
**Known difficult DL**		Airtraq	McGrath
		Bonfils	V-MAC
		Bullard	
		CTrach	
		GlideScope	
		Pentax AWS	
**Failed DL**		Airtraq	Bullard
		Bonfils	V-MAC
		CTrach	
		GlideScope	
		McGrath	
		Pentax AWS	

## Discussion

### The choice of devices to study

When considering the wide variety of airway devices currently available, it is impossible to perform a systematic review of all. With this is mind we limited our review to videolaryngoscopy and applied a rigid, objective inclusion criterion (that of at least 10 publications in the last 5 years) in an attempt to make the selection contemporary. No performance assessment of such a diverse group of devices will ever be perfect, but we have attempted to limit through our objective inclusion criteria those devices that have received the most recent development and where competition exists between different versions of similar equipment. The dynamic nature of the field is illustrated by the decision to discontinue the manufacture of the CTrach by LMA North America in December 2009 during the period of the review. However, the CTrach is still in clinical use and fulfilled the study inclusion criteria so remained in this systematic review. The main technique excluded from this review is flexible fiberoptic bronchoscopy. We feel this method has a specific clinical application and is difficult to compare with standard direct laryngoscopy. The inclusion criteria limited the choice of devices to the GlideScope, V-MAC (including C-MAC and Storz Berci DCI), Bullard, McGrath, Bonfils, Airtraq, Pentax AWS, LMA CTRACH. The recently introduced CMAC and the older Storz DCI and V-MAC were considered versions of the Storz Macintosh video laryngoscope for the purpose of this review and referred to as the V-MAC (video Macintosh) for the remainder of this review.

**Figure 2:**
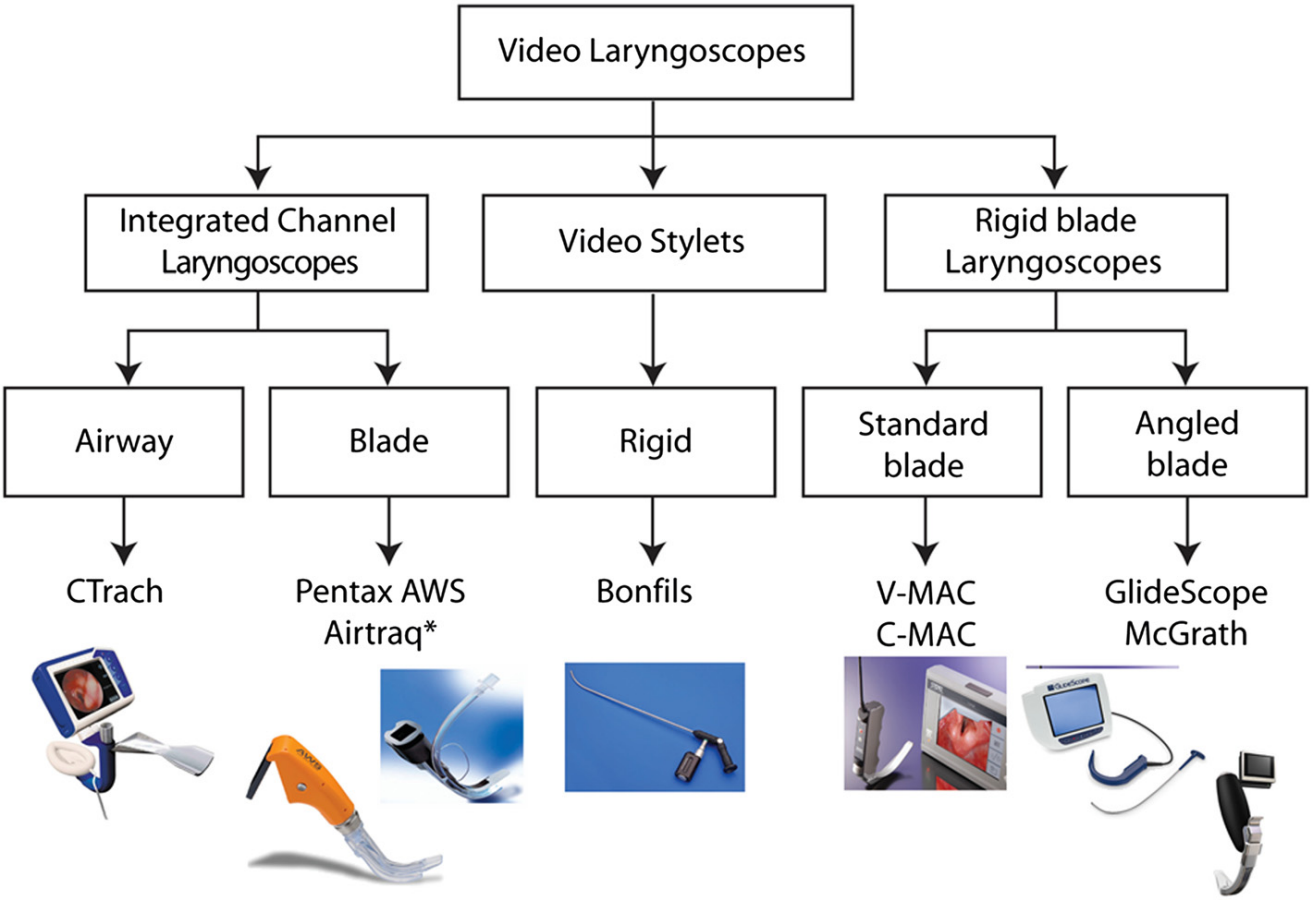
**A classification of videolaryngoscopic devices.** CTrach image courtesy of LMA North America. Pentax AWS image courtesy of Ambu USA. Airtraq image courtesy of Prodol Meditec S.A**.** Bonfils and C-MAC Â©2012 Photo Courtesy of KARL STORZ Endoscopy-America, Inc. GlideScope image courtesy of Verathon, USA. The McGrath series 5 image courtesy of Aircraft Medical, UK.

The devices returned by our methodology are presented in the following diagram (Figure 2). We have classified the videolaryngoscopes according their principle shape and form:

1. Presence of an integrated channel (to guide placement of the endotracheal tube).

2. The form of a videostylet (with the endotracheal tube placed around the device).

3. A rigid blade laryngoscopes (without a channel, the endotracheal tube requiring some kind of independent stylet to guide placement).

Rigid blade laryngoscopes are sub-divided into those with a “standard” blade and those with an angled blade as classified by Niforopoulou and colleagues [8]. There may be differences between the two types with respect to the glottic view on laryngoscopy and ease of intubation. Overall, we feel this classification scheme adds some clarity to the set of devices under examination. Clearly there are design differences between each device within these broad groups (for example presence of antifogging device etc.) Our classification is not novel as previous classifications of videolaryngoscopy have already been published [9,11] but use different criteria to differentiate.

### Subject classification

#### Predicting difficult direct laryngoscopy

This low incidence of difficulty encountered at direct laryngoscopy makes the study of true difficulty problematic. Our standard airway examination is a poor predictor of its occurrence [11,97]. The data from a meta-analysis of 50,760 patients found that the positive predictive value (PPV) of the Mallampati score (at predicting a Cormack and Lehane view 3 or greater) to be 16% [10]. Less than 1 in 5 of those subjects with a “positive” Mallampati score 3 or 4 actually had a Cormack and Lehane view of 3 or greater when DL was performed. Another study has suggested an even lower PPV for the Mallampati score [98]. Consequently any study that solely uses the Mallampati classification to determine difficulty will have a very low incidence of true difficulty encountered at endotracheal intubation. The combination of the limited power of our current airway assessment methods to predict difficult direct laryngoscopy, with the multiple definitions of difficulty makes the subject classification a potential source of controversy. Our criterion for difficult laryngoscopy was a Cormack and Lehane view 3 and above; the definition provided by the ASA in the Practice Guidelines for Management of the Difficult Airway (2003) [99]. We accept that this is a conservative estimate of difficulty, but this criterion actually returned relatively few articles specifically examining this finding at laryngoscopy.

The level of evidence table (Table 2) was compiled by dividing the articles into four groups:

1. **Unselected:** this group of articles included subjects considered to have normal airways based on their airway examination and risk factors for difficult laryngoscopy. Even though this group contains a number of subjects that were proven during the study to have true difficult laryngoscopy, the outcomes were reported for the group as a whole and not specifically for this often-small subgroup preventing their specific outcomes to be analyzed.

2. **At Higher risk of difficult direct laryngoscopy:** these articles included only subjects suspected to have an increased likelihood of difficult intubation because of one or more airway assessment test results or the presence of obesity (BMI > 35) or cervical spine limitation (collar or MILS). The data were reported for all those subjects fulfilling these criteria, irrespective of whether they were found to be good or poor direct laryngoscopic views during the study. As direct laryngoscopy was not performed before the videolaryngoscopy attempt the subjects had an unknown incidence of true difficult direct laryngoscopy (C&L >/= 3).

3. **Difficult direct laryngoscopy** (C&L >/= 3): These articles included subjects with a documented Cormack and Lehane view III or greater on direct laryngoscopy before the intervention.

4. **Failed direct laryngoscopy:** These articles included subjects upon whom direct laryngoscopy failed to achieve tracheal intubation.

### Quality assessment of the evidence

Before overall recommendations could be made regarding the efficacy of particular methods of VL, a measure of the quality of each study was made. This is particularly difficult regarding VL as the published studies consist of a mixture of observational (case–control and case series) with few actual randomized, controlled studies. The Agency for Healthcare Research and Quality performed a review of the many methods for assessing the quality of studies and found few that could be applied to both prospective randomized and observational studies [100]. Following their recommendations the current review used the methodology developed by the Scottish Intercollegiate Guidelines Network [12]. This method allowed non-analytical studies (eg. Case reports and case series) to contribute to the overall evidence (although at a much weaker score). Using defined criteria the methodological quality of each analytical study was made to give a quality rating (++ = good, + = adequate, - = poor) (Table 4). Of note, the SIGN methodology does not allow a quality assessment to be made for non-analytical studies. Each study was reviewed by 2 investigators (DH, OM) and entered into a standard data extraction table (Table 1). Where disagreement was found, this was discussed and consensus attained. Of note, a single study may appear multiple times in the data extraction table if multiple devices were investigated and data reported for each device. Evidence for one method of VL over another was presented as a level of evidence (Table 4) and then tied to a grade of recommendation in the discussion of these findings based upon the SIGN criteria [12] (Table 5).

**Table 4:** Levels of evidence

	
1++	RCTs with a very low risk of bias (or high quality meta-analyses, systemic reviews of RCTs)
1+	RCTs with a low risk of bias (or well conducted meta-analyses, systematic reviews of RCTs)
1-	RCTs with a high risk of bias (or meta-analyses, systematic reviews or RCTs)
2++	High quality case–control or cohort studies with a very low risk of confounding/bias/chance and a high probability that the relationship is causal (or High quality systematic reviews of case–control or cohort studies)
2+	Well conducted case–control or cohort studies with a low risk of confounding/bias/chance and a moderate probability that the relationship is causal
2-	Case–control or cohort studies with a high risk of confounding/bias/chance and a significant risk that the relationship is not causal
3	Non-analytic studies, eg. Case reports, case series
4	Expert opinion

Reproduced from Harbour R, Miller J. A new system for grading recommendations in evidence based guidelines. BMJ (Clinical research ed 2001;323:334-6) with permission from BMJ Publishing Group Ltd. RCTs, Randomized controlled trials.

**Table 5:** Grades of recommendations

	
**A**	At least one metanalysis, systematic review, or RCT rated as 1++ and directly applicable to the target population, or A systematic review of RCTs or a body of evidence consisting principally of studies rated as 1+ directly applicable to the target population and demonstrating overall consistency of results
**B**	A body of evidence including studies rated as 2++ directly applicable to the target population and demonstrating overall consistency of results, or extrapolated evidence from studies rated as 1++ or 1+
**C**	A body of evidence including studies rated as 2+ directly applicable to the target population and demonstrating overall consistency of results or extrapolated evidence from studies rated as 2++
**D**	Evidence level 3 or 4, or extrapolated evidence from studies rated as 2+

Reproduced from Harbour R, Miller J. A new system for grading recommendations in evidence based guidelines. BMJ (Clinical research ed 2001;323:334-6) with permission from BMJ Publishing Group Ltd.

### Review of the evidence

#### The evidence for efficacy of videolaryngoscopy

The performance of a device when compared with direct laryngoscopy relies on three main outcomes: overall success, 1st time success, and time to successful intubation. Glottic view is a desirable outcome but intubation can remain successful and timely despite a limited view of the glottis, and in the case of VL a good laryngeal view does not ensure successful intubation. After careful review of the literature it was decided that little could be summarized or deduced from the Time to Intubation; this outcome being so variably defined between the studies as to make it useless as a form of comparison. We left the summary data from the outcome in the tables for completeness. Instead, we focused on overall success and first time success when compiling our evidence and recommendations, supplemented with information regarding the attainment of glottic view when possible. We followed the methodology developed by the Scottish Intercollegiate Guidelines Network [12] which was recommended by the Agency for Healthcare Research and Quality when assessing the strength of evidence provided by both prospective randomized and observational studies [100].

#### Evidence for the use of video laryngoscopy in unselected patients

As previously discussed, the success rate for standard direct laryngoscopy in a general, unselected population without airway pathology is likely to be greater than 95% [10]. It must be noted that there is a difference between an improvement in laryngoscopic success (ie. achieving a view of the glottis) and success of intubation. Direct laryngoscopy is often successful, despite an inadequate view of the glottis. The review of videolaryngoscopy revealed an overall success rate for unselected patients of between 94 to 100% for all of the devices, which is similar to the high success rate of direct laryngoscopy. If used to lower the incidence of difficult intubation, videolaryngoscopy may have little to offer in this unselected patient population due to the low incidence of actual difficulty encountered. However, as we will later discuss, failure can occur during any intubation attempt and the utility of video laryngoscopy must be considered as an alternative intubation device when direct laryngoscopy fails. The performance benefit of videolaryngoscopy as an educational tool was not examined in the current review, but our opinion is that the techniques of videolaryngoscopy should be practiced in a normal population, and competency demonstrated, before attempting to use in a difficult laryngoscopic scenario. There is no current evidence to suggest an increased rate of traumatic airway complications compared with direct laryngoscopy in unselected patients although there are many case reports detailing injuries and hypotheses for their causation [101-108]. The lack of evidence for a particular device should not be interpreted as evidence against its use, but rather a weakness of the published evidence.

#### Evidence for the use of videolaryngoscopy in patients assessed to be at high risk of difficult direct laryngoscopy

When examining overall success the current review demonstrates a high rate of success when using the Airtraq, CTrach, GlideScope, Pentax AWS, and V-MAC videolaryngoscopes supported by level 1+ evidence (good prospective). There is weaker level 3 evidence (case series) to support the use of the Bonfils and Bullard. We found no evidence for the use of the McGrath in this clinical setting. Additionally, the review revealed level 1+ evidence (good prospective) for a higher proportion of Cormack and Lehane grade I views (compared to direct laryngoscopy) when using the Airtraq, CTrach, GlideScope, Pentax AWS, and V-MAC. The review revealed no evidence for the Bonfils, Bullard, and McGrath for the attainment of a higher proportion of C&L grade I views (compared to direct laryngoscopy).

Given the above evidence, for those patients judged to be at risk of having a difficult laryngeal view on direct laryngoscopy we recommend the use of the Airtraq, CTrach, GlideScope, Pentax AWS, and V-MAC, by an operator with reasonable prior experience, to maintain overall success at intubation and increase the likelihood of Cormack and Lehane grade I views compared to direct laryngoscopy (grade A recommendation) based on the SIGN criteria [12] (Table 5). Such selection does not preclude the possibility of awake intubation in accordance with the ASA Practice Guidelines for Management of the Difficult Airway (2003)[99].

#### Evidence for the use of videolaryngoscopy in difficult direct laryngoscopy (C&L >/= 3)

The current review demonstrates a high level of overall success when using the Airtraq, Bonfils, Bullard, CTrach, Glidescope, and Pentax AWS videolaryngoscopes supported by weak level 3 (case series) evidence. We found no evidence for success for the McGrath or V-MAC in this clinical setting. There is additional weak non-analytic evidence (level 3) to suggest that the use of the CTrach, GlideScope, and Pentax AWS results in an increased percentage of Cormack and Lehane grade I views of the glottis. This is in broad agreement with the previous review by Mihai et al. [11].Given the above evidence, for those patients with known difficulty direct laryngoscopy (C&L view III or IV) we cautiously recommend the use of the Airtraq, Bonfils, Bullard, CTrach, GlideScope, and Pentax AWS (grade D recommendation) by an operator with reasonable prior experience, to maintain the overall success rate of intubation based on the SIGN criteria [12]. This particular recommendation must be considered with respect to the current ASA guidelines that recommend the use of a technique which maintains spontaneous ventilation if at all possible, in patients with known or predicted difficult laryngoscopy.

#### Evidence for the use of videolaryngoscopy as a rescue device after failed direct laryngoscopy

After failure of initial direct laryngoscopy morbidity has been shown to increase when more than two attempts are made at laryngoscopy during emergency intubations performed beyond the operating room [109]. Perhaps, given this finding, the Difficult Airway Society of the United Kingdom suggest in their failed intubation guideline, that a provider makes no more than 2 attempts with the same device before moving on to an alternative laryngoscopic device, with the maximum number of laryngoscopic attempts limited to 4 [110]. The ASA guidelines currently do not define the maximum number of attempts with a particular device [99] but suggest that consideration be made to the use of an alternative intubation device if the primary device fails. The videolaryngoscopes would seem to fulfill the requirement of an alternative intubation device if an anesthesia provider is skilled in their use, and the device exhibits a high 1st attempt success rate. The current review demonstrates a high level of overall success, following failed intubation via direct laryngoscopy, when using the Airtraq, Bonfils, CTrach, Glidescope, McGrath, and Pentax AWS videolaryngoscopes supported by weak level 3 evidence (case series). We found no evidence for success for the Bullard or V-MAC in this clinical setting. There is additional weak level 3 evidence (case series) for a high first attempt success rate with use of the Bonfils and Pentax AWS in this setting. There is supplemental weak non-analytic evidence (level 3) to suggest that the use of the Airtraq, CTrach, McGrath, and Pentax AWS results in an increased percentage of Cormack and Lehane grade I views of the glottis after failed direct laryngoscopy.

Given these findings we recommend use of the Airtraq, Bonfils, CTrach, GlideScope, McGrath, and Pentax AWS, used by an operator with reasonable prior experience, as an alternative intubation device following failed direct laryngoscopy (grade D recommendation) based on the SIGN criteria [12]. There may be extra reason to consider use of the Bonfils or Pentax AWS given their high 1st attempt success in this setting (grade D recommendation).

### The limitations of the current review

#### Classification using the Mallampati as the sole predictor of difficulty during direct laryngoscopy

The use of the Mallampati classification as the predictor of difficulty at direct laryngoscopy is an oversimplification. We have presented Shiga’s work demonstrating that it is a very poor predictor of difficulty alone even when combined with other preoperative airway assessments. Unfortunately, the various predictors of difficulty at DL are variably presented in the literature, with Mallampati being the only consistently performed preoperative test. Studies examining patients with cervical spine limitation and obesity were included into the “at higher risk of difficulty group” as the authors and publishers considered these subjects to be at higher risk of difficulty.

#### Grading the view at laryngoscopy

It is clear the ability of a laryngoscopic device to produce a good view of the glottis is a desirable characteristic of such a device. To allow some comparison between devices we considered a Cormack and Lehane grade I view of the glottis to be beneficial irrespective of whether it is obtained by direct or indirect means. This measure allows comparison between studies as a Cormack and Lehane grade I view is reliably recorded irrespective of whether the standard Cormack and Lehane, the various forms of modified Cormack and Lehane, or the Percentage of Glottic Opening is used in its assessment. Unfortunately the other grades of laryngoscopic view (grade 2, 2a, 2b etc.) are so variably recorded as to make other comparisons impossible. The limitation of using such a strict measure of glottic view improvement is the risk missing a lesser, but perhaps clinically significant improvement in glottic view afforded by device use, for instance an improvement from a grade 3 to a 2a view of the glottis.

The concept of using the Cormack and Lehane classification when comparing direct laryngoscopy with the variety of methods of videolaryngoscopy is questionable. These grading schemes are designed and validated for direct laryngoscopy only; however, this measure is used throughout all of the studies, as no other alternate scheme exists. The actual difficulty in tube passage during videolaryngoscopy (unlike direct laryngoscopy) is often independent of the view obtained on the screen. Therefore, the description of the view found during videolaryngoscopy as a simple Cormack and Lehane view analogous to that found during direct laryngoscopy may be inappropriate as it doesn’t necessarily correlate with success. We suggest that during videolaryngoscopy a grading scheme that incorporates the difficulty encountered during passage of the endotracheal tube should be used. One simple method of grading this would be to describe the difficulty (easy, difficult, or failed) with a record of the glottic view obtained (modified Cormack and Lehane) followed by the name of the device. Difficulty could be defined by the performance of multiple attempts or the use of airway adjuncts to place the tube. For example, if the procedure is difficult but ultimately successful then this could be reported as a “Difficult Grade II GlideScope; rescued with the use of a tracheal tube introducer”. This information would allow decisions to be made if the use of a videolaryngoscope is contemplated at a later date, but also allow the different types of videolaryngoscopes to be more easily compared.

#### Device variability and the comparison of efficacy

These devices are “moving targets”, i.e. new designs are continually introduced and existing designs are modified. This makes studies of older designs sometimes of questionable applicability to those currently being sold. We considered the devices in the current review to not have changed in form or function to an appreciable amount in the study period. Where new videolaryngoscope blade shapes were introduced, but failed to fulfill the inclusion criteria, they were excluded from the analysis (example the CMAC “D blade” and King Vision™). The differences between the devices extend toward other design features, such as the presence of a heating element to prevent fogging of the view etc. These differences cannot be easily described in a simple classification scheme.

#### Operator performance and competency

Experience level and competency of the operators performing laryngoscopy was not presented or accounted for during analysis in any of the studies in the review. Instead the studies generally stated that the operators were appropriately trained and experienced in the procedure. Indeed, a simple expression of an operator’s number years in practice or number of previous successful intubations doesn’t provide a measure of the competency of a that operator in the use of a particular device. This is a serious limitation of the studies included in this review, which limits our conclusions and applicability of our recommendations. The current review was limited to device performance in appropriately experienced users. An improvement in the success of novices with the use of these devices was beyond the scope of this review. Any recommendations for their use must be considered in this context, and in the decision-making associated with a well-considered airway management strategy.

#### Risk of bias within studies

When assessing the quality of randomized controlled trials particular emphasis is placed on the quality of the randomization process and blinding of subject and observer. Of these 2 factors, blinding is especially hard to address in a study design investigating videolaryngoscopy, and is generally poorly performed in the literature resulting in a universally poor score with respect to blinding. We found no article to which we could award the 1++ level of evidence class (excellent prospective). Randomized controlled trials may not be the best method of assessing the management of rare outcomes (such as true difficult laryngoscopy or intubation) or where blinding of operator to the device under study is impossible.

#### Risk of bias across studies

Current methods to assess the quality of available evidence, outlined by the Agency for Healthcare Research and Quality (AHRQ) [100], are generally characterized as weighting their quality measures heavily towards randomized controlled trials. Relatively few of the methods suggested by the AHRQ actually allow observational retrospective studies to be included in any level of evidence summary. It is perhaps here where retrospective review of high quality outcome data in large databases generated by perioperative Anesthesia Information Management Systems (AIMS) can be particularly useful. Like many topics this subject likely suffers from publication bias and selective reporting within studies.

## Conclusion

In conclusion, we describe a field of research limited by poor subject classification and variable outcomes. We used a reasoned scientific approach to clarify and quantify the strength of evidence to support the use of some modern videolaryngoscopic devices. We found overall limited evidence of efficacy for many of the videolaryngoscopic devices. However, our review allowed us to produce the following limited recommendations: Firstly, in patients assessed to be at higher risk of difficult laryngoscopy we recommend the use of the Airtraq, CTrach, GlideScope, Pentax AWS, and V-MAC to achieve successful intubation (Grade A recommendation [12]). Secondly, in difficult direct laryngoscopy (Cormack and Lehane view III or IV on direct laryngoscopy) we cautiously recommend the use of the Airtraq, Bonfils, Bullard, CTrach, GlideScope and Pentax AWS to achieve successful intubation (Grade D recommendation [12]) used in accordance with the ASA practice guidelines for management of the Difficult Airway. Thirdly, additional evidence exists to recommend the use of the Airtraq, Bonfils, Bullard, CTrach, Glidescope, McGrath, and Pentax AWS following failed direct laryngoscopy to achieve successful intubation (Grade D recommendation [12]). Additional consideration should be made to use of the Bonfils and Pentax AWS given the evidence for 1st attempt success in this setting (Grade D recommendation). Future investigation would benefit from the precise qualification of study group airway characteristics, the use of consecutive rather than unselected subjects, the measurement and standardization of operator competency, the blinding of observers, and the standardization of outcome measures. These steps would reduce bias and help interpretation and metanalysis.

Financial Support: All funding was solely from departmental sources. No external funding was solicited or used.

## Competing interests

The authors have no conflicts of interest.

## Authors’ contributions

DH designed the review, extracted the data, summarized the findings, and composed the manuscript. OM extracted the data, summarized the findings, and contributed to the manuscript. DH summarized the findings. SK participated in the study design, reviewed the manuscript and coordinated the team efforts. All authors read and approved the final manuscript.

## Implication statement

This systematic review of the efficacy of videolaryngoscopy in orotracheal intubation classifies the patient groups under study into four clinical entities: unselected, at higher risk of difficulty, difficult direct laryngoscopy, and failed direct laryngoscopy. The evidence of efficacy is presented and recommendations are made.

## Pre-publication history

The pre-publication history for this paper can be accessed here:

http://www.biomedcentral.com/1471-2253/12/32/prepub
